# Effect of physical stimulation (gingival massage) on age-related changes in gingival microcirculation

**DOI:** 10.1371/journal.pone.0233288

**Published:** 2020-05-20

**Authors:** Satoko Wada-Takahashi, Ko-ichi Hidaka, Fumihiko Yoshino, Ayaka Yoshida, Masahiro Tou, Masato Matsuo, Shun-suke Takahashi

**Affiliations:** Department of Oral Science, Graduate School of Dentistry, Kanagawa Dental University, Yokosuka, Kanagawa, Japan; University of Mississippi Medical Center, UNITED STATES

## Abstract

The decline in circulatory function with aging may be alleviated by a combination of gingival massage (physical stimulation) and mechanical cleaning. Several studies have reported the systemic effect of physical stimulation on various parts of the body, including its therapeutic effect on pain in the neck and shoulders that becomes evident with age, and improvement in blood circulation. In contrast, few studies have reported on the changes in gingival microcirculation induced by gingival massage, while no previous study has evaluated the effect of gingival microcirculation on age-related changes in the hemodynamics of the oral cavity. This study aimed to investigate how gingival massage affects age-related changes in gingival microcirculation. Male Wistar rats (7-week, 6-month and 1-year old) were prepared for a gingival massage group and a control group. Mechanical stimulation was applied on the maxillary molar gingiva for 5 seconds twice a week for 4 weeks. Subsequently, gingival reactive hyperemia was measured using a laser Doppler flowmeter. In addition, morphological analyses were also performed by hematoxylin and eosin and Indian ink staining and a vascular resin cast model. Base Flow, maximum response (Peak), and time required for the maximum response to halve (T_1/2_) were reduced in 1-year-old rats compared with the other age groups. In the mechanical stimulated group, T_1/2_ was increased in 7-week, 6-month, and 1-year-old rats, and total blood flow (Mass) was increased in 6-month and 1-year-old rats. In addition, clear blood vessel networks and loop-like revascularization were only observed in the mechanical stimulated group. Changes in age-related decline in gingival microcirculatory function and vascular construction were reported in this study, and the results suggested that gingival massage activates both the functional and morphological aspects of gingival microcirculation and may be effective for maintaining oral health.

## Introduction

Several studies have reported on the relationship between aging and conditions such as hypertension, heart disease, and lifestyle diseases [1, 2]. Periodontal disease is included in these lifestyle diseases related to circulation, and in the early stages, circulatory dysfunction occurs through endothelial dysfunction of peripheral blood vessels [3]. People tend to unconsciously touch, rub, massage, or apply pressure to these areas with peripheral circulatory failure or to areas with pain or discomfort caused by circulatory failure [4]. These actions are known as massage and are performed in different areas of the body [4, 5].

Whole body massage is one of the most effective and widely practiced alternative therapies [5–8]. Several studies have reported on the various effects of massage therapy. Massage increases weight in preterm infants via a mechanism different from that of exercise [6]. Massage is also effective in relieving neck and shoulder pain, and this effect is reported to increase with the frequency of the massage [7, 8]. In the oral cavity, the relationship between periodontal disease and gingival blood flow has been studied. Early studies using experimental ischemic models showed no change in gingival blood flow caused by periodontal disease [9]. On the other hand, it has been reported that changes in factors that could affect tissue circulation were observed due to ischemia [10, 11]. Furthermore, it has been reported that blood flow changes according to the severity of periodontitis [12]. Combining gingival massage with conventional approaches, such as removal of the bacteria that causes periodontal disease through mechanical cleaning, may effectively treat periodontal disease, whose incidence increases with age [5].

The effects of massage can be attributed to muscle relaxation, balance between muscles in the joints, mechanical changes in the muscles, increased joint flexibility and proprioception, increased lymph circulation, changes in immune and inflammatory responses, improved sleep, and interruption of pain signals [13–20]. Numerous studies have evaluated the effects of massage on the vagal nerve and the entire body [6, 21]. The expected effect of massage on exercise capacity is due to increased circulating blood volume. In fact, massage is often performed immediately after exercise to promote blood circulation [22].

Blood flow in the gingiva reduces with age [23]. Conversely, brushing increases blood flow [24]. An experiment using dogs reported that mechanical stimulation was effective in improving the gingival microcirculatory function [25]. However, no previous study has performed a functional and morphological analysis of the effect of massage on the microcirculation adjustment mechanism in the field of oral medicine. Furthermore, no study has reported on the effect of massage on age-related changes in oral hemodynamics.

Laser Doppler flowmetry (LDF) can evaluate microcirculatory function in human and animal tissue. This method is noninvasive, easy to use, and has been used to evaluate the circulatory function of soft tissues since 1980 [26]. LDF has been used to detect gingival reactive hyperemia (GRH) as an index of microcirculatory function [27–30]. As advocated by Doppler, LDF is performed based on the following principle: “vibrational energy radiated by a moving object or reflected from an object changes the vibration frequency proportional to the relative speed of the object [31]”; this theorizes that a fast-moving object changes frequency to a greater extent than a slow-moving object. LDF uses laser light that oscillates at a known frequency as its energy source to measure the velocity of red blood cells. Blood vessels contain heterogeneous red blood cells moving at different speeds, and the incident light frequency changes relative to the velocity of the blood vessels. Rapidly moving red blood cells have large changes in frequency, while slow moving red blood cells have small changes in frequency. The proportion of light with changing frequency that returns to the receiver is proportional to the number of red blood cells, while the size of the change in frequency is proportional to the blood flow velocity. Therefore, theoretically, the blood flow can be calculated from the sum of the number of red blood cells and blood flow velocity [32].

Reactive hyperemia (RH) is characterized by a transient increase in blood flow observed when the arterial blood inflow to organs or tissues is temporarily blocked and then re-perfused, and this reaction occurs above preischemic levels. RH is an endothelium-dependent vasorelaxation response and is an indicator of vascular endothelial cell function and peripheral microcirculation adjustment function [29]. We used this method to conduct a noninvasive analysis of microvascular function in the mouth [29, 30, 33, 34]. Moreover, we previously conducted a study to determine the relationship between microcirculatory dynamics in the oral cavity and systemic hemodynamics [29, 30]. In this study, we aimed to analyze the age-related changes in gingival microcirculation using 7-week-old, 6-month-old, and 1-year-old rats with this GRH as an index to investigate the effect of physical stimulation (gingival massage).

## Materials and methods

### Experimental animals

In this study, we attempted to minimize the distress experienced by the test animals by performing only the required tests. This study was approved by the Kanagawa Dental University Animal Experiment Ethics Committee (approval numbers: 16–056, 16–057, 16–064, 17–001, 17–002, 17–016, and 17–017). All experiments were conducted in accordance with the Kanagawa Dental University Animal Experiment Guidelines.

This study used male Wistar rats (7-week-old: 191.1±4.5–289.2±9.1 g, 6-month-old: 386.3±7.9–418.8±11.1 g, 1-year-old: 470.0±10.7–503.7±12.7 g; data are shown as the mean ± standard error of the mean) (Japan SLC, Shizuoka, Japan). The rats were raised in a room with controlled illumination (12-hour light and dark cycles) and temperature (22±3°C), with 3–5 animals per cage, and were provided with *ad libitum* access to food and water. Rats in individual age groups (7-week-old, 6-month-old, and 1-year-old) were divided into the control and gingival massage groups (MSG group), with 6–10 animals in each group [29, 30, 33].

### Gingival massage

**Fig 1:**
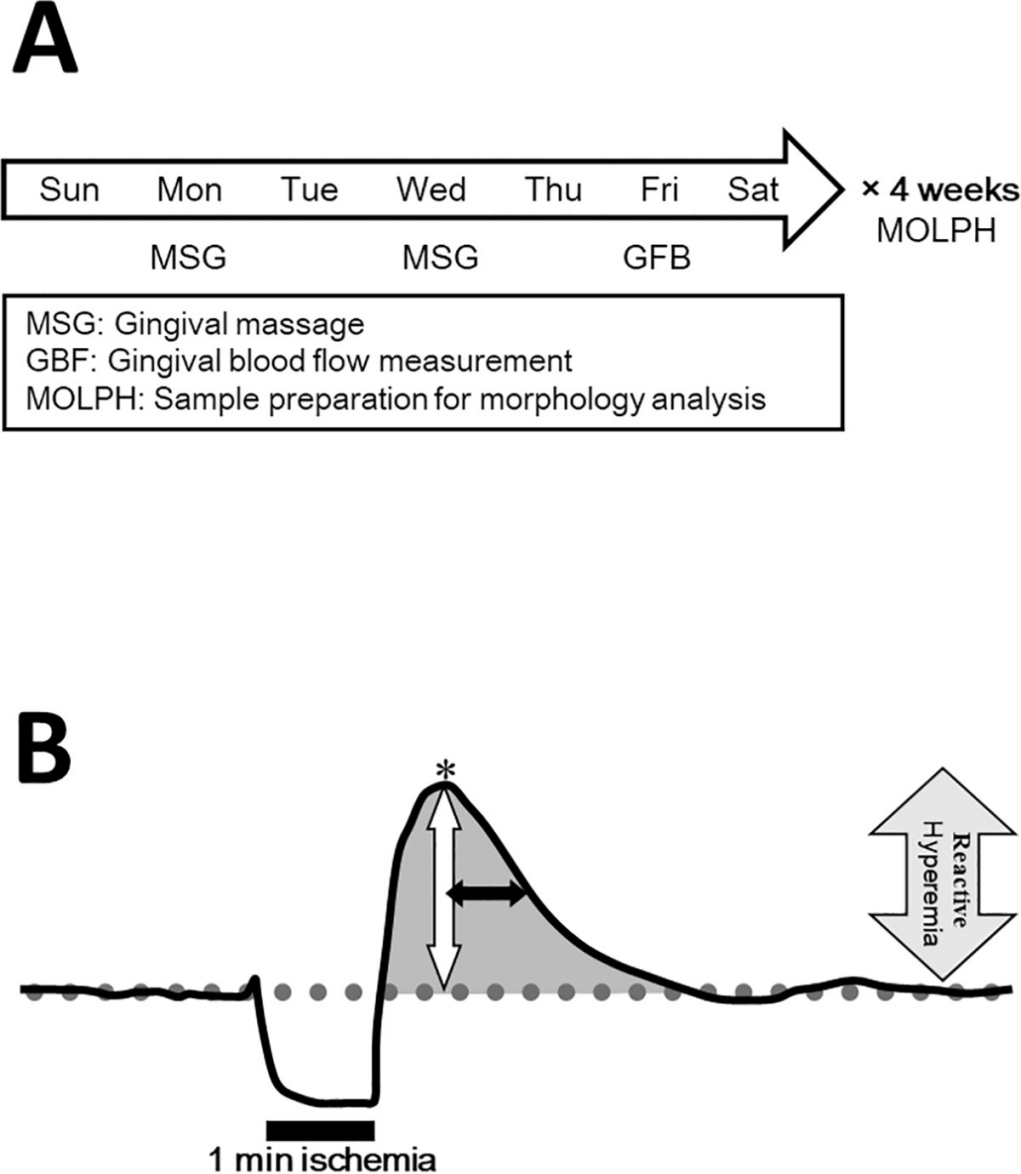
Experimental design. (A) The experiment schedule. Gingival massage was performed on the gingiva on the mesial aspect of the maxillary left first molar twice a week for 4 weeks, for a total of eight sessions. The blood flow velocity in the same location was measured 3 days after the gingival massage using a laser Doppler flowmeter. After completing 4 weeks of blood measurements, hematoxylin and eosin + Indian ink staining specimens and resin injected blood vessel mold specimens were prepared. (B) The typical change in gingival blood flow during reactive hyperemia. Base blood flow (dotted line: Base Flow), the difference between the Base Flow and the peak blood flow* (white two-way arrow: Peak), time required until the peak blood flow halves (black two-way arrow: T_1/2_), and total increase in blood flow (gray shaded area: Mass).

All rats in each age group were anesthetized with inhalation isoflurane, and anesthesia was maintained at a concentration of 2.5% [29, 30]. After measuring body weight, the rats were placed in a dorsal position on a warmed bench (30 × 45 cm) maintained at 37°C. All four limbs were fixed with adhesive tape at a 45-degree angle relative to the midline, and the lower and upper jaw incisors were fixed in an open position with kite twine [29]. Gingival massage was performed on the gingiva on the mesial aspect of the maxillary left first molar for 5 seconds with a stimulation pressure of 5–10 gf twice a week. Pressure was measured by a force transducer. Gingival massage was applied manually by the same person (SWT). The experimental period lasted for 4 weeks, and a total of eight gingival massage sessions were performed (Fig 1A). A GUM electric toothbrush (TS-45, Sunstar, Osaka, Japan) and a high-speed reversing-type dedicated head (number of rotations, approximately 2,500 rpm, Sunstar, Osaka, Japan) with a prophy cup (#1805: point screw type, Eicoh, Tokyo, Japan) cut to a diameter of approximately 2.0 mm attached to the tip were used for gingival massage. The control groups were only subject to inhalation anesthesia with isoflurane. The gingival massage pressure measurements were obtained by connecting the aforementioned GUM electric toothbrush to a force transducer interface and adaptor, and the force measurements were recorded on a monitor (PCD-400A, UI-55A, Kyowa Electronic Instruments, Tokyo, Japan). The data were analyzed using an analysis software (DCS-100A, Kyowa Electronic Instruments, Tokyo, Japan).

### Gingival blood flow measurements

After the rats were anesthetized with inhalation isoflurane using the same method used during gingival massage, the four limbs of the rat were fixed to a warmed bench. The blood flow in the gingiva on the mesial aspect of the maxillary left first molar (gingival blood flow, GBF) was measured using a blood flow measurement probe (diameter 2.0 mm) attached to a laser Doppler flowmeter (LDF, TBF-LN1, Unique Medical, Tokyo, Japan) [29]. All GBFs were estimated with this laser Doppler probe, and calibration was performed before each analysis by the automatic calibration system according to the manufacturer’s instructions. GRH was induced using a blood flow measurement probe by applying pressure at 10–20 (ml/min/100g) to the gingiva at the massage site for 1 minute and releasing it. GRH was evaluated using the indices of base blood flow (Base Flow), the difference between the Base Flow and the peak blood flow* (white two-way arrow: Peak), time required for peak blood flow to halve (T_1/2_), and total increase in blood flow (Mass) (Fig 1B). The heart rate was monitored at the same time to determine the effect of GRH on cardiac function. The measured output signal was recorded on a computer hard disk via an A/D converter and displayed simultaneously on a monitor. The recorded GBF was analyzed using data analysis software (Chart v. 8.1.8, AD Instruments, Colorado, U.S.) [29].

### Morphological analysis

#### Preparing hematoxylin and eosin staining and Indian ink staining

The rats from each group were bled under pentobarbital sodium (45 mg/kg, intraperitoneally) anesthesia by perfusing 0.2% heparin in saline into the ascending aorta. The tissue was fixed and perfused in 10% neutral buffered formalin solution to maintain the vascular lumens [35]. After perfusing ink into the blood vessels, the maxilla was excised, hard tissue was decalcified using hydrochloric acid, and the specimens were embedded in paraffin to obtain 50-μm-thick sections. Hematoxylin and eosin staining was then performed using normal methods. The ink infusion state in the blood vessels was observed under an optical microscope [36]. Blood vessels stained with Indian ink were evaluated using 200 × 200 μm sections using NIH ImageJ software (https://imagej.nih.gov/ij/).

All morphological images in this study showed typical results obtained from the same series of preparations.

#### Preparing vascular corrosion casting models of gingival circulation

Blood vessel corrosion cast specimens were prepared after completion of the blood flow measurement experiment to observe the changes in the three-dimensional structure of the microvascular network.

The rats from each group were bled under pentobarbital sodium (45 mg/kg, intraperitoneally) anesthesia by perfusing 0.2% heparin in saline into the ascending aorta. The tissue was fixed and perfused in 10% neutral buffered formalin solution to maintain the vascular lumens. After fixing, synthetic resin (Mercox2, Ladd Research industries, Vermont, U.S.) was injected into the blood vessels. The specimens were immersed in 5% sodium hypochlorite solution to decompose the soft tissue. All specimens were washed with warm water (40°C) and then freeze-dried. After coating the specimens with platinum-palladium (JFC-1200, JEOL, Tokyo, Japan,) they were observed using a scanning electron microscope (JSM6301F, JEOL, Tokyo, Japan) [37]. The morphological images in this paper all showed typical results.

### Reagent

The following drugs were used in the experiment: isoflurane (MSD Animal Health, Tokyo, Japan), pentobarbital sodium (Kyoritsu Seiyaku, Tokyo, Japan), and heparin sodium (EA Pharma, Tokyo, Japan).

### Statistical analysis

To compare the differences between two groups, Bonferroni post-hoc comparisons were used following one-way analysis of variance (ANOVA). A two-way ANOVA was used to compare the differences among more than three groups, followed by Bonferroni post-hoc comparisons. Data are expressed as mean ± standard error of the mean. A *P* value lower than 0.05 was considered significant. Microsoft Excel 2013 (Microsoft, WA, USA) and GraphPad Prism 6 (GraphPad Software, CA, USA) were used for the data analyses.

## Results

### Evaluation of vascular response with GRH

GBF was immediately reduced in all blood flow measurements by applying pressure to the gingiva using the probe, and this action had no effect on cardiac function. GRH was observed when pressure was released after applying gingival pressure for 1 minute (Fig 1B).

**Fig 2:**
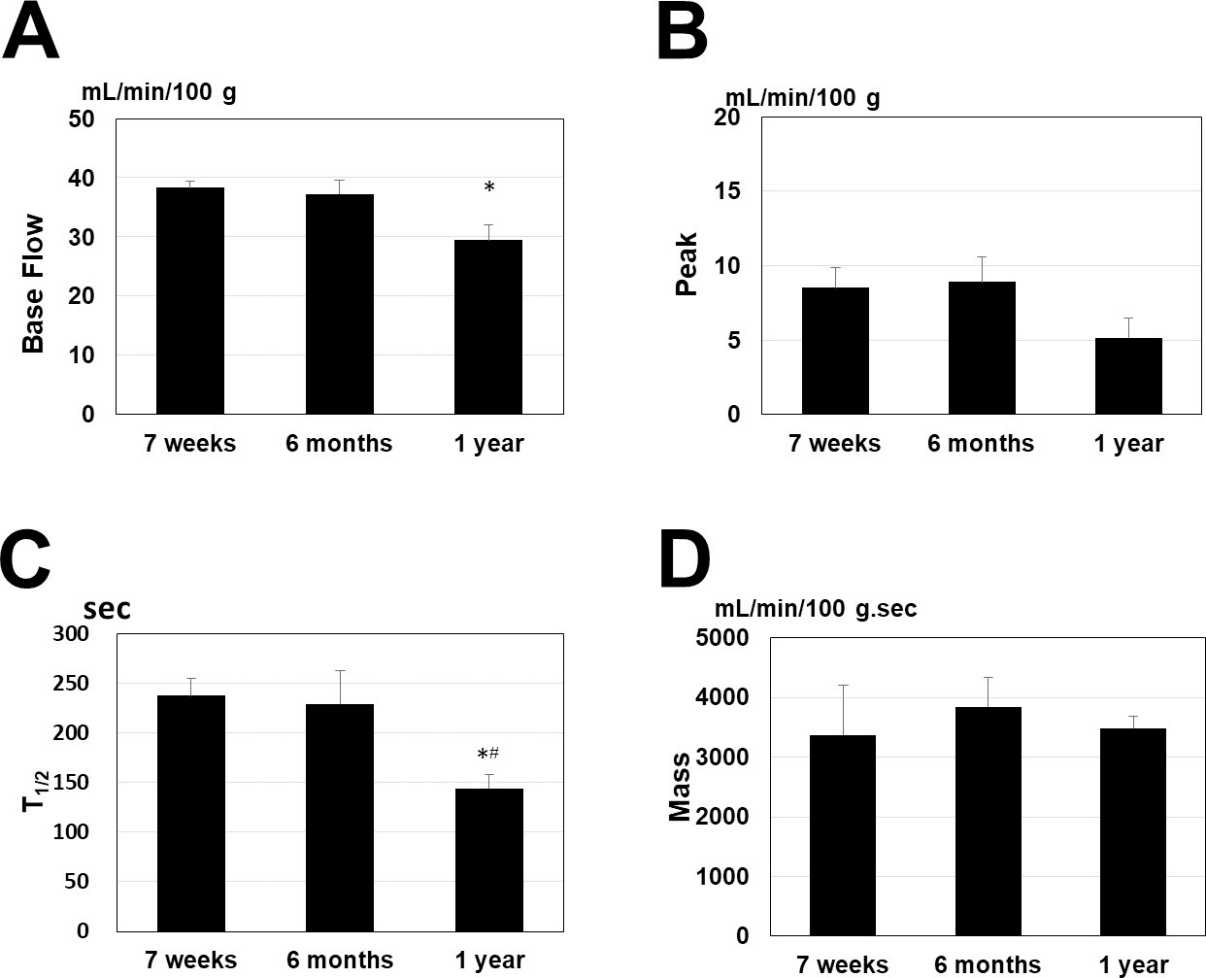
Changes in age-related reactive hyperemia. (A) Base Flow, (B) Peak, (C) T_1/2_, (D) Mass. The 1-year-old rats showed reduction in Base Flow compared with the 7-week-old rats as well as a shortening of T_1/2_ compared with the 7-week-old and 6-month-old rats. The data are expressed as mean ± standard error of the mean (n = 8–10). *P<0.05 vs. 7 weeks, ^#^*P*<0.05 vs. 6 months.

**Fig 3:**
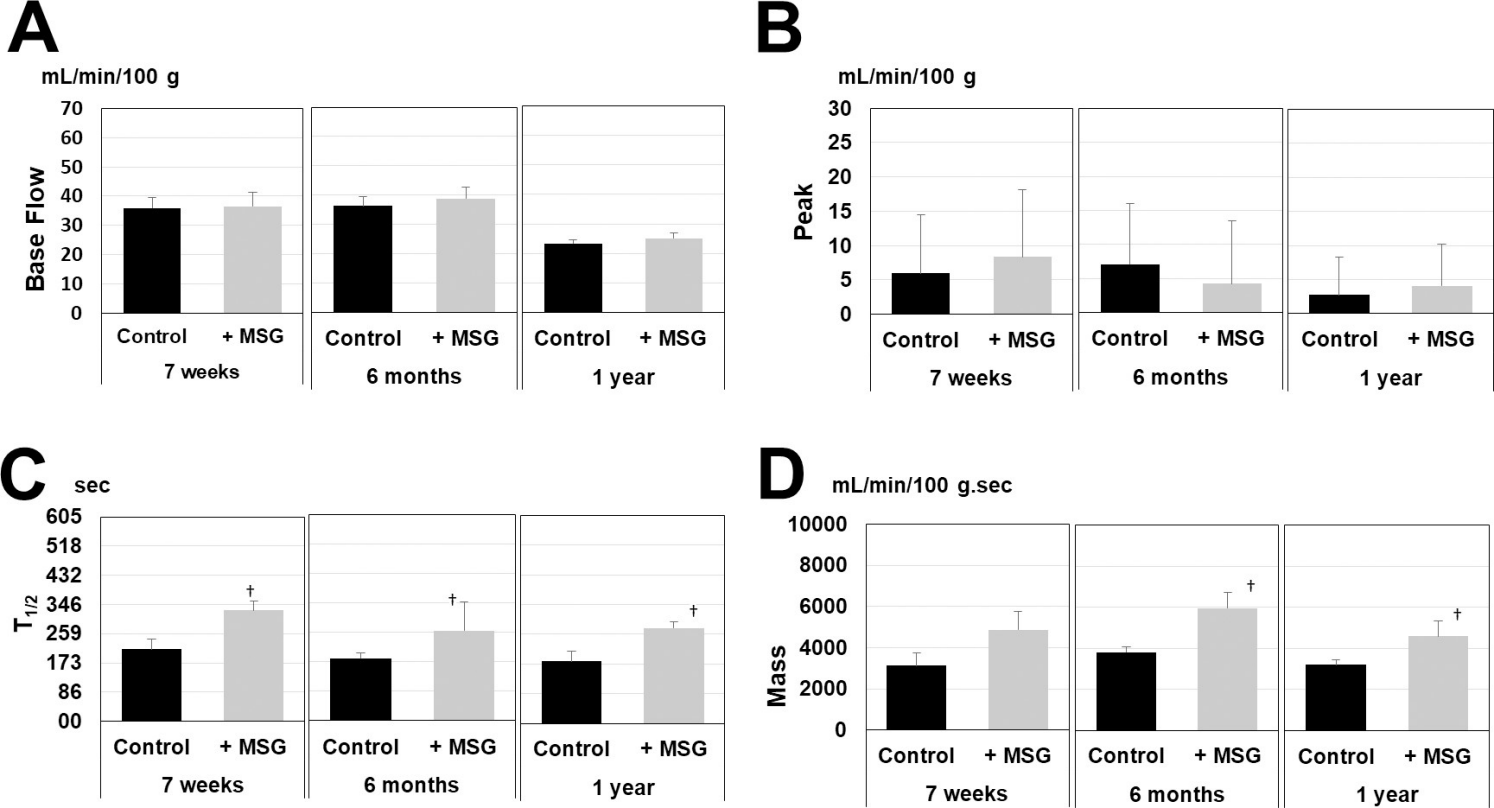
Effect of gingival massage on age-related decline in reactive hyperemia. (A) Base Flow, (B) Peak, (C) T_1/2_, (D) Mass. Compared with the control group, 4 weeks of massage in the gingival massage group lengthened the T_1/2_ in the 7-week-old rats and increased the mass in the 6-month-old and 1-year-old rats. The data are expressed as mean ± standard error of the mean (n = 6). *P<0.05 vs. each control groups.

Comparison of age-related changes in GRH was performed between each of the age groups in the control group. The 1-year-old rats had reduced Base Flow compared with the 7-week-old rats; moreover, the Peak showed a tendency to decrease, and the T_1/2_ was shortened in the 1-year-old rats compared with the 7-week-old and 6-month-old rats (Fig 2A, 2B and 2C). The control group and MSG group were compared. The results showed that T_1/2_ lengthened in the 7-week-old, 6-month-old, and 1-year-old rats with repeated massage over 4 weeks and that the Mass increased in the 6-month-old and 1-year-old rats (Fig 3C and 3D). A two-way ANOVA revealed no interaction between aging and MSG with regard to Baseflow, Peak, T_1/2_ and mass. The results of the two-way ANOVA were as follows: Baseflow: df = 2, F = 3.316, p = 0.852; Peak: df = 2, F = 3.316, p = 0.176; T_1/2_: df = 2, F = 3.316, p = 0.896; Mass: df = 2, F = 3.316, p = 0.937). In other words, each age group benefits similarly from massage.

### Morphological findings in hematoxylin and eosin and Indian ink staining specimens

**Fig 4:**
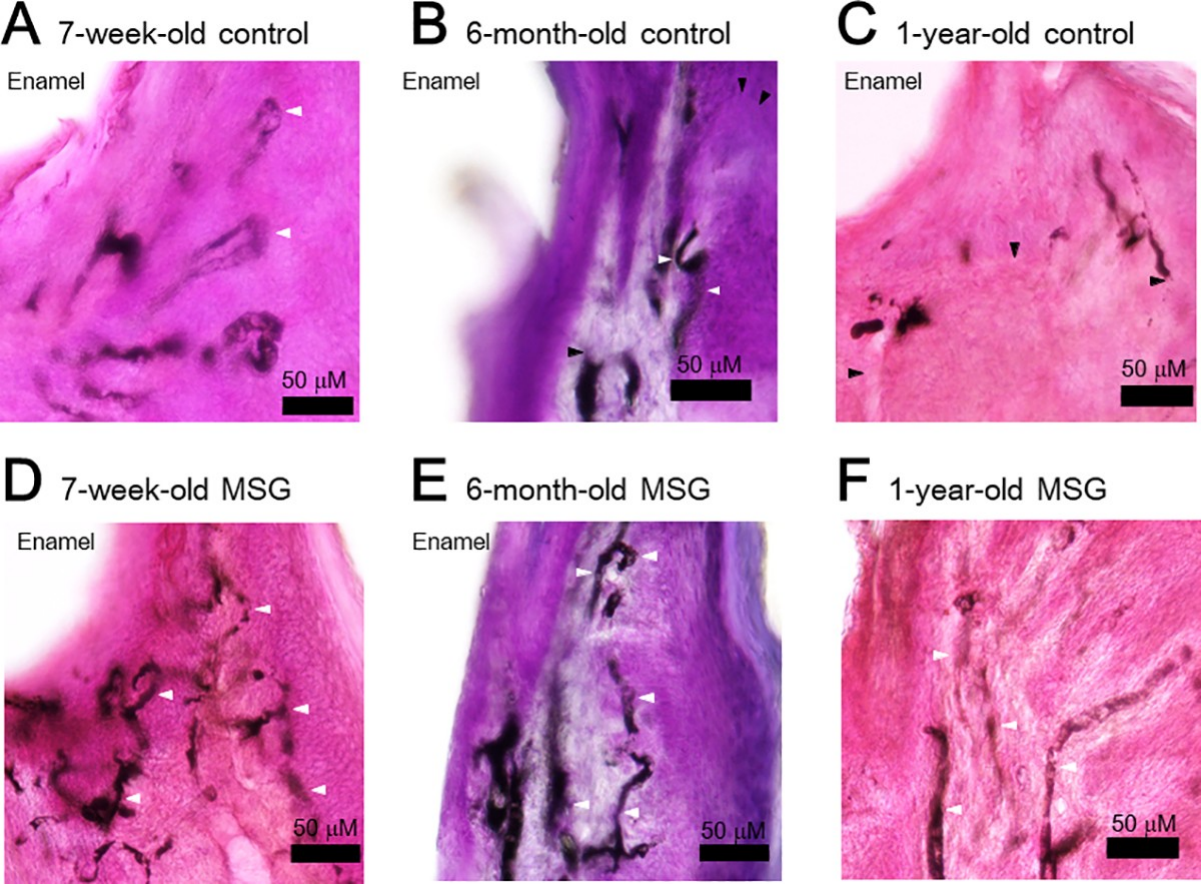
Morphological analysis (hematoxylin and eosin + Indian ink staining specimens). (A–C) Control group, (A) 7-week-old, (B) 6-month-old, (C) 1-year-old, (D–F) Gingival massage (MSG) group, (D) 7-week-old, (E) 6-month-old, (F) 1-year-old. Looped blood vessels were observed corresponding to the lamina propria under the outer epidermis of the gingival surface layer. In the control group, the height of the looped blood vessels (white arrowheads) reduced with age. There was interruption of blood flow in some blood vessels; hence, the ink was unable to enter these vessels (black arrowheads). Compared with the control group, most of the blood vessels in the MSG group maintained an ink-injected circulation (white arrowheads). The total area of blood vessels stained with ink within a certain range of images (200×200 μm) was increased in the MSG group compared to the control group for all three ages (A: 4177.0±1199.9, B: 2180.0±527.3, C: 1858.2±781.3, D: 5285.4±602.2, E: 3759.8±1592.0, and F: 3878.2±657.5 μm^2^, n = 3 in each group) (A: 4177.0±1199.9, B: 2180.0±527.3, C: 1858.2±781.3, D: 5285.4±602.2, E: 3759.8±1592.0, and F: 3878.2±657.5 μm^2^, n = 3 in each group). Regarding the one-year-old rats, there was a significantly higher amount of blood vessel staining with ink in the gingival massage group compared to the control group.

In the 7-week-old control group, looped blood vessels were observed corresponding to the uneven gingival lamina propria located immediately under the outer epithelium of the gingival surface layer (Fig 4A, white arrowheads). The vascular network was also observed immediately under the adhesion epithelium on the left side of the figure. In the 6-month-old control group, the height of the looped blood vessels was reduced (Fig 4B, white arrowheads), and the ink was disrupted in some areas (Fig 4B, black arrowheads). In the 1-year-old control group, the looped blood vessels observed in the 7-week-old group were almost completely flattened (Fig 4C, white arrowheads), and several blood vessels exhibited circulatory disturbance with the disruption of the ink (Fig 4C, black arrowheads). In all age groups, the MSG group had a higher blood vessel density with the ink-injected circulation maintained than the control group (Fig 4D–4F, white arrowheads).

### Morphological findings in the vascular corrosion casting model of gingival circulation

**Fig 5:**
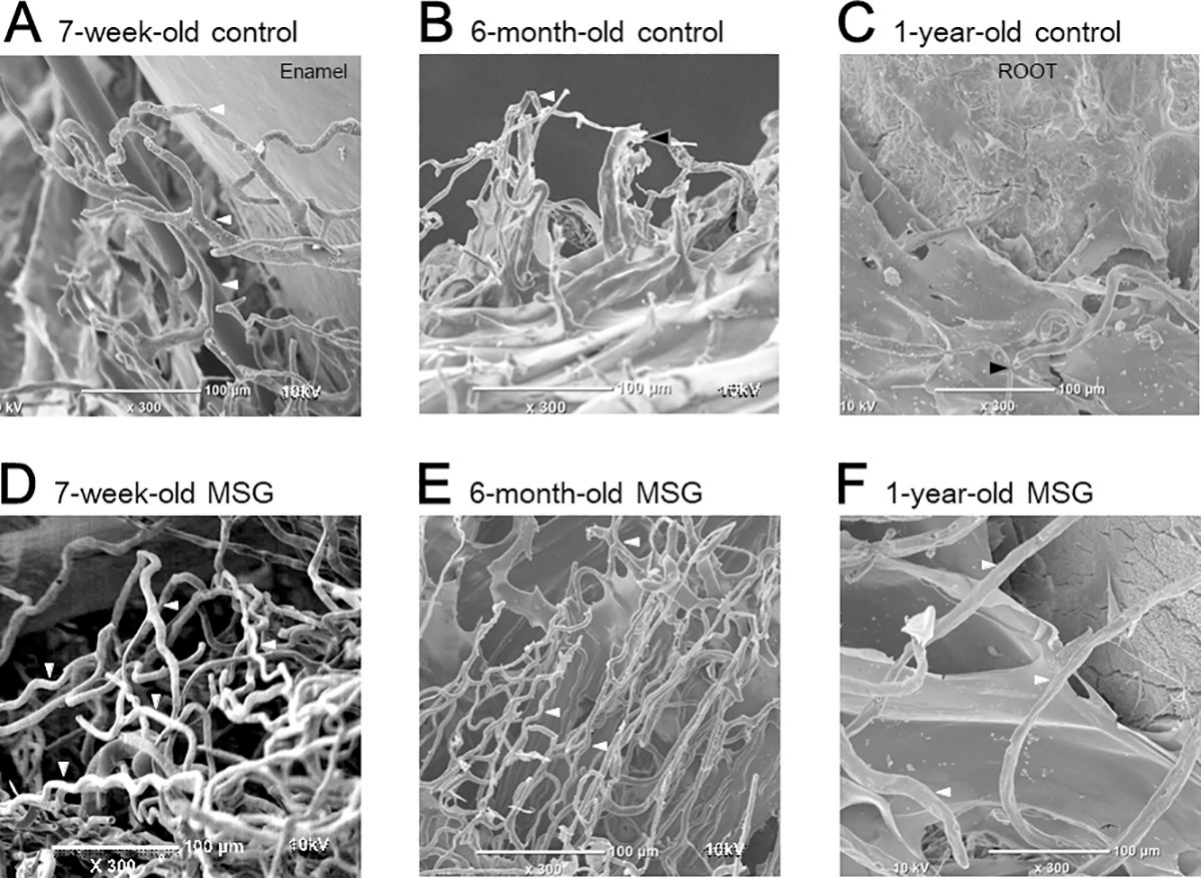
Morphological analysis (vascular corrosion casting model of gingival circulation). (A–C) Control group, (A) 7-week-old, (B) 6-month-old, (C) 1-year-old, (D–F) Gingival massage (MSG) group, (D) 7-week-old, (E) 6-month-old, (F) 1-year-old. This photograph shows the synthetic resin injected into the blood vessel. Therefore, where there is blood flow (or vasculature), there is resin, and where there is no blood flow, there is no resin. The 7-week-old control group (A) has blood flow (vasculature) in the looped capillaries around the teeth, indicating that blood flow decreases with age. In the massage group, there was an increase in blood flow (vasculature) compared to the control group at each period. In the control group, the loops (white arrowheads) had age-related reduction in height and exhibited loss of loop morphology. Part of the surface of the blood vessels was rough; the flow of blood in some blood vessels was interrupted (black arrowheads). The MSG group had a dense vascular network including loops (white arrowheads) compared with the control group.

Vascular corrosion casting models of gingival circulation were observed under a three-dimensional image observation microscope, and the images observed were similar to those of the Indian ink staining sections. Highly looped blood vessels were observed surrounding the tooth cervix in the 7-week-old group (Fig 5A, white arrowheads). In the 6-month-old group, the height of the loops had declined. Moreover, there was loss of regularity (Fig 5B, white arrowheads) and blood flow interruption (Fig 5B, black arrowheads). In the 1-year-old group, a number of vascular networks surrounded the tooth apex, the loop shape was lost, and there was blood flow interruption (Fig 5C, black arrowheads). Rough surface blood vessels were also observed in the control group (Fig 5A, 5B and 5C). This structure showed morphological changes in the vascular lumen, and these changes increased with age.

In the MSG group, the 7-week-old rats had a high blood vessel density, including loops, indicating recovery of circulation (Fig 5D, white arrowheads). In the 6-month-old rats, the height of the loops was lower than that of the 7-week-old MSG group, but there was a dense vascular network (Fig 5E, white arrowheads). In the 1-year-old rats, although the height of the loops had decreased and had changed to a flat vascular network, there were obvious blood vessels compared with the control group (Fig 5F, white arrowheads).

## Discussion

RH results in the regulation of local circulation and reoxygenation of ischemic tissue [34]. It is known to be involved in various vascular regulatory functions, including metabolic, myogenic, and physical functions [37]. In medicine, flow-mediated dilatation and plethysmography using forearm RH are widely used as vascular function evaluation methods with RH as an index, and these are used to predict the risk of developing lifestyle diseases, among others, or to evaluate symptoms [38, 39]. According to a study conducted by Ishibashi et al., smokers have reduced vascular endothelial cell function compared with non-smokers [39]. The microvasculature distribution in the gingiva may be damaged as a person ages. Therefore, measurement and analysis of the microvascular function in the oral cavity have been conducted using this method with RH as an index [29, 30]. In this study, we evaluated the vascular endothelium-dependent relaxation of blood vessels, with GRH as the indicator, to determine the age-related changes in hemodynamics of the oral cavity and improvements induced by physical stimulation (gingival massage).

The results of our functional analysis of age-related RH changes with LDF showed reduced vascular responsiveness in 1-year-old rats (Fig 2A and 2C). A number of angiopathies, including reduced vascular endothelium-dependent relaxation, have been reported by previous studies conducted in older patients and aged animals [40, 41]. Reduced vascular endothelium-dependent relaxation in aged animals is considered to be caused by factors such as reduction in the number of receptors for vasodilation of vascular endothelial cells [42], reduction in the production of nitric oxide (NO) [40], and decreased guanylate cyclase activity in vascular smooth muscle cells [43]. The results of this study also showed age-related reduction in vascular endothelial function, but these changes are considered to be due to age-related reduction in the expression of endothelial nitric oxide synthase (eNOS) protein and reduced NO. Zhang et al. reported that the production of reactive oxygen species (ROS) from macrophages increases with age, and this promotes oxidative stress [44]. Previous studies have shown that once oxidative stress increases, vascular endothelial function declines [29]. We have confirmed that the superoxide anion radical (O_2_^**·**−^) is involved in this decline in vascular endothelial function [29].

It would be assumed that that the age-related decline in vascular endothelial function shown in this study may have been caused by the consumption of NO and by increasing age-related O_2_^**·**−^. In the vascular corrosion casting model of gingival circulation, some blood vessels presented with a rough structure (Fig 5A, 5B and 5C). This structure showed morphological changes in the vascular lumen and may suggest the occurrence of microangiopathy. There was also age-related interruption of blood flow observed in the hematoxylin and eosin + Indian ink staining specimens and vascular corrosion casting model of gingival circulation (Fig 4B and 4C, black arrowheads; Fig 5B and 5C, black arrowheads). This interrupted blood flow was considered to be due to reduction in the vascular relaxation response caused by age-related decline in NO. As indicated above, the results of the morphological analysis also suggested a reduction in the vascular relaxation response.

The results of functional analysis of age-related decline in RH showed that the 4-week gingival massage inhibited the decline in vascular responsiveness (Fig 3C and 3D). This is considered to be due to the shearing stress added to the vascular endothelial cells with gingival massage and the increased blood flow (reperfusion) due to the endothelial-dependent vascular relaxation response mediated by NO. Shearing stress is defined as a tangential friction force acting on the blood vessel walls due to blood flow [45]. It has been reported that shearing stress also activates eNOS [46,47]. There may have been an increase in the production of NO derived from vascular endothelial cells in the MSG group compared with the control group. This is considered to be a result of increased in the production of NO due to the activation of eNOS caused by the shearing stress during gingival massage.

Conversely, in the hematoxylin and eosin + Indian ink staining specimens, there were more ink-injected blood vessels in the MSG group than in the control group (Fig 4D, 4E and 4F, white arrowheads) This result is supported by the results of the ImageJ analysis. In the vascular corrosion casting model of gingival circulation, there were more looped blood vessels in the MSG group than in the control group (Fig 5D, 5E and 5F, white arrowheads). Looped blood vessels are found in healthy gingiva [35], and increase in the number of looped blood vessels in the MSG group suggests that gingival massage improved blood vessel morphology. The increased density of blood vessel distribution is considered to be a result of the vascular relaxation response due to an increase in the production of NO and resumption of blood flow in the blood vessels. As indicated above, the morphological analysis also revealed the effect of gingival massage on age-related gingival microcirculation.

The time for gingival massage in this study was set based on the study conducted by Matsumoto *et al*. [48]. When Matsumoto et al. stimulated one location for 5 seconds and 10 seconds using an electric gingival massager, it promoted gingival circulatory function. However, they reported that there was no significant difference between the group with 20 second stimulation and the group with no stimulation [48]. This report showed that the gingiva may be over-stimulated depending on the massage time. It also showed that massage performed for less than 10 seconds can improve the gingival circulation. In addition, recent studies have shown that reactive hyperemia after short-term compression (5 seconds only) in human gingiva lasts much longer than on the skin and spreads into a much wider area than the stimulated region, as shown via imaging [49]. This study also showed that a 5-second massage improved the function and morphology of gingival microcirculation among rats with age-related decline in gingival microcirculatory function and changes in vascular construction.

Furthermore, given that the effect of massage was observed in all age groups, it can be expected that gingival massage performed at a young age would maintain the function and morphology of healthy gingival microcirculation. Moreover, performing massage at an advanced age could help improve the gingival microcirculatory function and vascular construction. It is considered that gingival massage has an effect on the gingival microcirculation irrespective of when it was started and the age of the person, probably as a result of the activation of eNOS due to the shearing stress applied during gingival massage and the resulting increase in NO, which triggers a vascular relaxation response. This study also showed the effect of gingival massage on age-related gingival microcirculation. Further studies are warranted to determine the effect of gingival massage on age-related increase in O_2_^**·**−^.

## Conclusion

This study investigated the age-related changes in hemodynamics in the oral cavity and the effect of physical stimulation on age-related changes in gingival microcirculation. The results indicated that gingival microcirculatory function declines with age. Furthermore, based on morphological findings, several blood vessels developed various circulatory disorders due to the interruption of blood flow, particularly in aged rat specimens. This study showed that physical stimulation improved age-related decline in gingival microcirculation. This finding indicates that functional and morphological changes can occur in the gingival microcirculation with age. It also suggests that physical stimulation (gingival massage) improves gingival microcirculatory function and morphology and is effective for maintaining oral health.

## Supporting information

S1 Data (XLSX)

S2 Data (XLSX)

S3 Data (XLSX)
